# Gallbladder Hydrops

**DOI:** 10.7759/cureus.18159

**Published:** 2021-09-21

**Authors:** Rhea Sharma, Thor S Stead, Ilya Aleksandrovskiy, John Amatea, Latha Ganti

**Affiliations:** 1 Emergency Medicine, Chantilly High School, Chantilly, USA; 2 Medicine, The Warren Alpert Medical School of Brown University, Providence, USA; 3 Emergency Medicine, Ocala Regional Medical Center, Ocala, USA; 4 Emergency Medicine, Lakeland Regional Health, Lakeland, USA; 5 Emergency Medicine, Envision Physician Services, Plantation, USA; 6 Emergency Medicine, University of Central Florida College of Medicine, Orlando, USA; 7 Emergency Medicine, HCA Healthcare, Orlando, USA

**Keywords:** abdominal ultrasonography, right upper quadrant pain, abdominal pain, hydropic gallbladder, gallbladder hydrops

## Abstract

The authors describe the case of a middle-aged female who presented to the emergency department with exquisite right upper quadrant pain. Computed tomography scan of the abdomen revealed a hydropic gallbladder, confirmed with abdominal ultrasonography. The clinical presentation, imaging findings, and management of this interesting condition are discussed.

## Introduction

Gallbladder hydrops, also known as hydropic gallbladder or mucocele of the gallbladder, occurs secondary to obstruction of the cystic duct, often by a gallstone. Gallstone disease is very common. In the United States (US) alone, approximately 14 million men and 6 million women between the ages of 20 to 74 have gallstones. Several factors such as age, obesity, and hormones contribute to the high prevalence of gallstones. A diagnosis of gallbladder hydrops can be made when the gallbladder is distended with mucus, water, or clear liquid content instead of bile. It is caused by the prolonged blockage of the cystic duct, usually by an impacted gallstone [1]. Patients who present with this condition experience acute or chronic cholecystitis, while most gallstones themselves are asymptomatic. In this case report, the authors describe the case of a patient who presented to the emergency department with progressive right-sided abdominal pain.

## Case presentation

A 50-year-old female presented to the emergency department with right-sided abdominal pain. The patient stated that three days prior to presentation, she had eaten some food that made her feel "queasy". She thought her symptoms could be food-related and she waited a few days to see if things would resolve on their own. When symptoms did not improve, she went to an urgent care facility that diagnosed her with a urinary tract infection and prescribed her nitrofurantoin. She started taking the antibiotics but still had the right upper quadrant pain. She suspected it might still be related to the food she ate, so she took some bismuth subsalicylate and simethicone. Both of these medications somewhat alleviated the pain, and she felt okay the day prior to the presentation. By night, however, the pain re-emerged, and by early morning on the day of presentation, the pain worsened enough for her to visit the emergency department. She denied any fevers, chills, chest pain, shortness of breath, nausea, vomiting, diarrhea, headache, or urinary symptoms. The patient does not smoke cigarettes, drink alcohol, or use marijuana. Her vital signs included a temperature of 98.5° F, pulse 85 beats per minute, respirations 18 breaths per minute, and blood pressure 132/60 mmHg.

Physical examination revealed a well-developed, well-nourished female in no acute distress. The only positive finding on the physical examination was right upper quadrant tenderness to palpation. Laboratory analysis revealed markedly elevated bilirubin at 2.1 mg/dL (Table 1).

**Table 1:** Patient’s laboratory values

Name of Lab	Reference Range	Value
Sodium	135 - 145 mmol/L	135
Potassium	3.5 - 5.3 mmol/L	3.7
Chloride	98 - 107 mmol/L	101
Carbon Dioxide	21 - 32 mmol/L	27
Blood Urea Nitrogen	7 - 18 mg/dL	12
Creatinine	0.6 - 1.3 mg/dL	0.9
Glucose	74 - 106 mg/dL	98
Lactic Acid	0.4 - 2.0 mmol/L	1
Calcium	8.4 - 10.2 mg/dL	8.9
Total Bilirubin	0.0 - 1.0 mg/dL	2.1 H
Aspartate Aminotransferase	15 - 37 Units/L	108 H
Alanine Transaminase	12 - 78 Units/L	168 H
Total Alkaline Phosphatase	45 - 117 Units/L	109
Total Protein	6.4 - 8.2 g/dL	8.1
Albumin	3.4 - 5.0 g/dL	4.5
Amylase	25 - 115 Units/L	81
Lipase	73 - 393 Units/L	29 L
White Blood Cell count	4.1 - 9.3 K/mm3	7.5
Red Blood Cell count	3.28 - 5.50 M/mm3	4.92
Hemoglobin	12.1 - 15.1 gm/dL	13.8
Hematocrit	35.5 - 46.9 %	42.8
Platelet Count	150 - 450 K/mm3	290
Mean Platelet Volume	7.3 - 10.9 um3	9
Absolute Basos (auto)	0.0 - 0.2 K/mm3	0.1
Nucleated Red Blood Cells %	0 - 0 %	0
Immature Granulocytes	0 - 0 K/mm3	0
Neutrophils #	1.4 - 6.5 K/mm3	3.9
Lymphocytes #	1.2 - 3.4 K/mm3	3
Monocytes #	0.1 - 0.6 K/mm3	0.5
Eosinophils #	0 - 0.7 K/mm3	0.1
Nucleated RBCs #	0 - 0 K/mm3	0
Urine Color	YELLOW	AMBER H
Urine Appearance	CLEAR	HAZY H
Urine pH	5.0 - 8.5	6
Urine Specific Gravity	///	1.025
Urine Protein	NEGATIVE mg/dL	TRACE
Urine Glucose (Stick)	mg/dL	NEGATIVE
Urine Ketones	NEGATIVE mg/dL	80 H
Urine Blood	NEGATIVE	NEGATIVE
Urine Nitrate	NEGATIVE	POSITIVE H
Urine Bilirubin	NEGATIVE	1+ H
Urine Urobilinogen	0.2 - 1.0 EU/dL	2.0 H
Urine Leukocyte Esterase	NEGATIVE	MODERATE H
Urine WBC	0 - 5 /Hpf	10 - 20 H
Urine Epithelial Cells	0 - 5 /Hpf	6 - 10
Urine Bacteria	NEGATIVE	MANY

**Figure 1:**
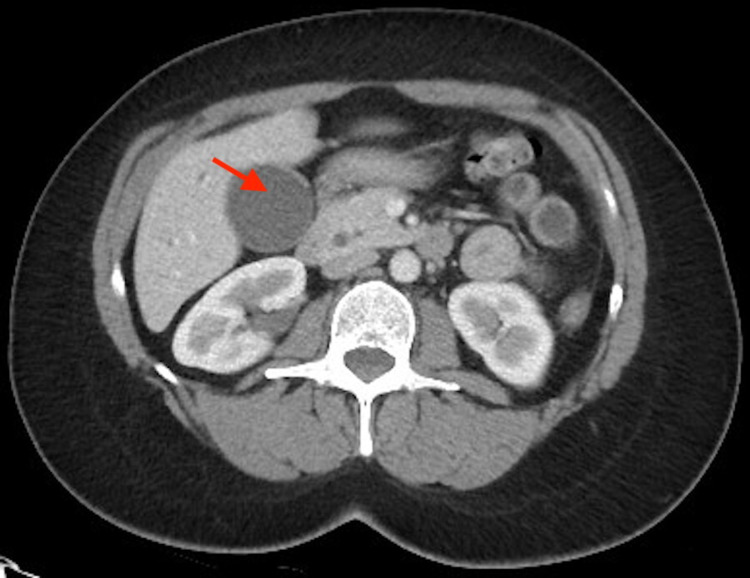
CT angiography scan demonstrating hydropic gallbladder on the left (red arrow)

Urinalysis suggested a urinary tract infection. CT scan revealed a hydropic gallbladder, with dilatation of the common bile duct and intrahepatic biliary tree possibly due to noncalcified stone or mass at the ampulla (Figure 1).

**Figure 2:**
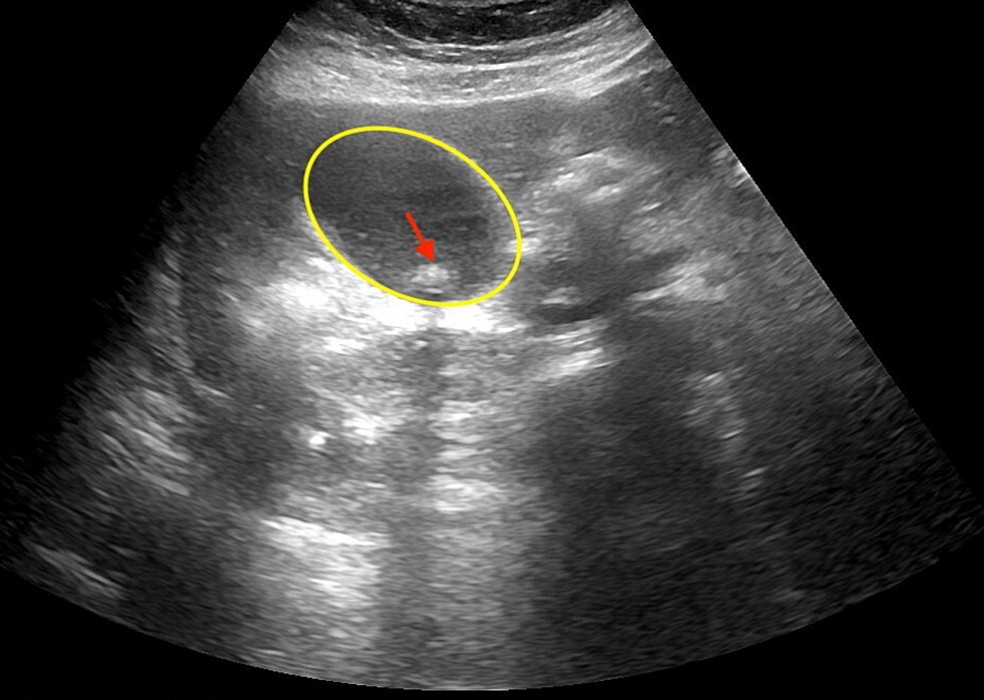
Abdominal USG demonstrating hydropic gallbladder (yellow oval) containing a gallstone (red arrow)

Right upper quadrant USG revealed gallstones and gallbladder sludge with mild diffuse intrahepatic and extrahepatic biliary dilatation, without sonographic criteria for acute cholecystitis (Figure 2).

The patient received 4 mg of intravenous morphine and 8 mg of intravenous ondansetron for pain and nausea. Her symptoms subsided and she was discharged with follow-up to gastroenterology.

## Discussion

**Figure 3:**
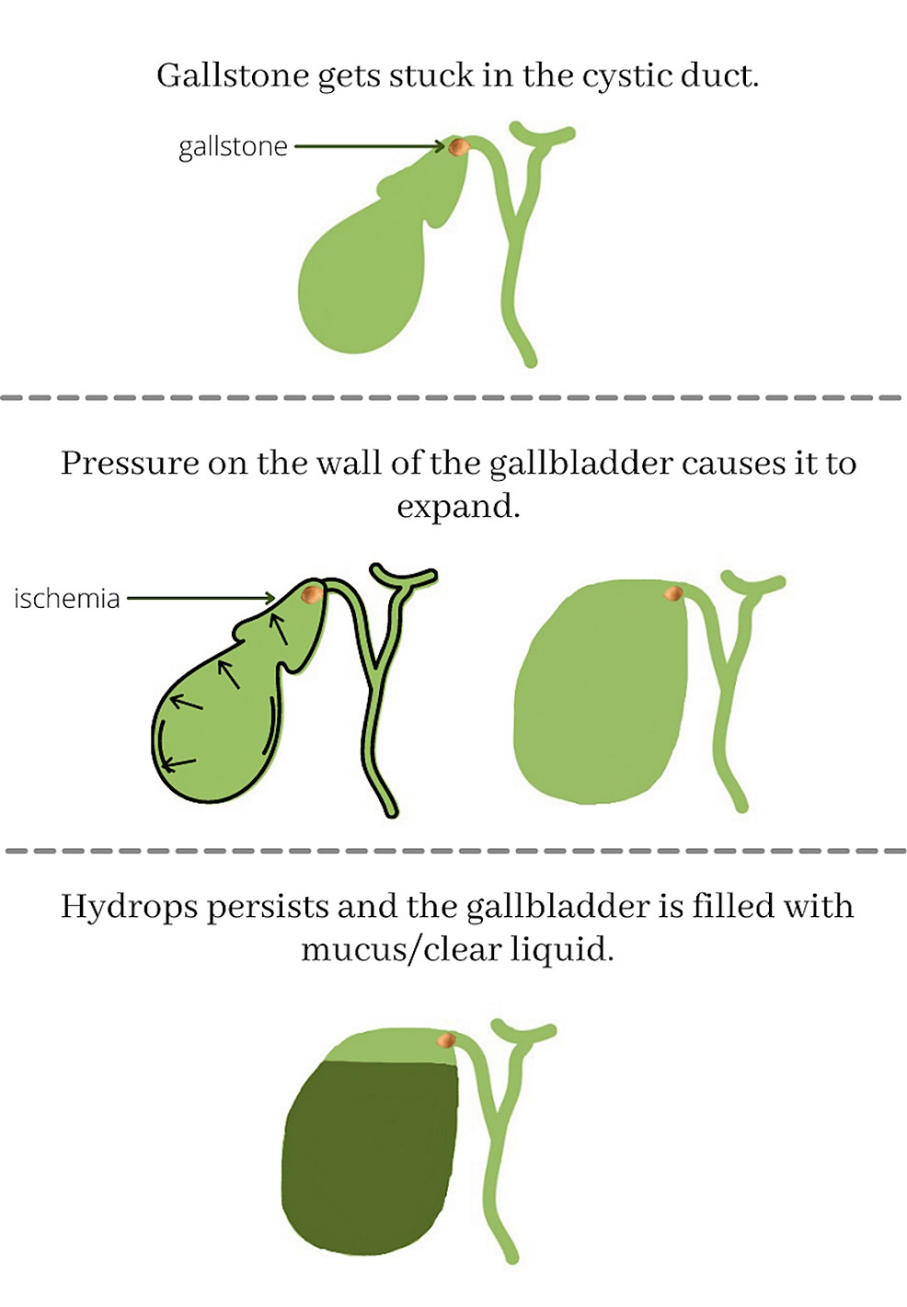
An illustration of the process by which mucus/other liquids form in the stretched gallbladder Created by Rhea Sharma on Canva

Gallbladder hydrops most often occurs when the cystic duct is blocked usually by a gallstone, causing an excessive amount of mucus, water, or other clear liquid (Figure 3) to accumulate.

The gallbladder stores and concentrates bile between meals. It largely contributes to the regulation of bile composition by several absorptive and secretory enzymes. Changes in gallbladder motor function not only can contribute to gallstones, but can also change the bile acid composition. If the gallbladder is incorrectly functioning, the bile may not completely be emptied, which may lead to gallstone formation. Gallstones can vary in size from being as small as a grain of sand to as large as a golf ball, and there can be one or many. Gallstones can get stuck in the neck of the gallbladder, the cystic duct. When this happens, the gallbladder mucosa reabsorbs the bile salts and over time be replaced by clear, watery mucus. This leads to pressure on the gallbladder walls causing the gallbladder to expand. Over time, the mucosa will be replaced by clear/watery mucus [1].

Hydropic gallbladder occurs in both men and women, and there are certain populations that are more prone to this condition than others. Studies have shown that a hydropic gallbladder is more likely in patients in their 40s, women/pregnant women, and obese patients. Drastic weight loss or acute illnesses may also increase the risk, such as post gastric bypass. Conditions that cause the breakdown of blood cells also increase the incidence of gallstones as does estrogen, which increases bile cholesterol. Consequently, women on birth control medication containing estrogen have double the likelihood of gallstone formation compared to men. Patients with chronic illnesses such as diabetes also have an increase in gallstone formation due to neuropathy [1].

The normal adult gallbladder is 7-10 cm in length and 3-4 cm in transverse diameter [2]. An enlarged gallbladder is considered inflamed; cholecystitis can lead to serious complications such as gallbladder rupture [3]. Tests to diagnose this condition include blood tests, USG (abdominal or endoscopic), CT scan, or a hepatobiliary iminodiacetic acid (HIDA) scan. Hydropic gallbladder often involves hospital treatment to control the inflammation in the gallbladder. Acute treatments include intravenous hydration, analgesia, and sometimes antibiotics. Often, the condition is recurrent, and will eventually require surgical management with cholecystectomy [4].

Approximately 300,000 cholecystectomies are performed annually in the US [5]. It can be performed either laparoscopically, which is less invasive, or sometimes as an open procedure. Complications of cholecystectomy include bile leak, bleeding, infections, and injuries to nearby structures [6].

## Conclusions

Understanding the importance and function of the gallbladder is important for clinical practice, as gallstones are a highly prevalent problem. Blockage of the gallbladder cystic duct can result in a hydropic gallbladder, as presented in this case report.
